# A novel scalable, robust downstream process for oncolytic rat parvovirus: isoelectric point-based elimination of empty particles

**DOI:** 10.1007/s00253-016-8071-x

**Published:** 2017-01-14

**Authors:** Barbara Leuchs, Veronika Frehtman, Markus Riese, Marcus Müller, Jean Rommelaere

**Affiliations:** German Cancer Research Center Tumor Virology F010, Im Neuenheimer Feld 280, 69120 Heidelberg, Germany

**Keywords:** H-1 parvovirus, pI, Empty capsid elimination, Chromatography, CIM^®^ column, Upscaling

## Abstract

The rodent protoparvovirus H-1PV, with its oncolytic and oncosuppressive properties, is a promising anticancer agent currently under testing in clinical trials. This explains the current demand for a scalable, good manufacturing practice-compatible virus purification process yielding high-grade pure infectious particles and overcoming the limitations of the current system based on density gradient centrifugation. We describe here a scalable process offering high purity and recovery. Taking advantage of the isoelectric point difference between full and empty particles, it eliminates most empty particles. Full particles have a significantly higher cationic charge than empty ones, with an isoelectric point of 5.8–6.2 versus 6.3 (as determined by isoelectric focusing and chromatofocusing). Thanks to this difference, infectious full particles can be separated from empty particles and most protein impurities by Convective interaction media^®^ diethylaminoethyl (DEAE) anion exchange chromatography: applying unpurified H-1PV to the column in 0.15 M NaCl leaves, the former on the column and the latter in the flow through. The full particles are then recovered by elution with 0.25 M NaCl. The whole large-scale purification process involves filtration, single-step DEAE anion exchange chromatography, buffer exchange by cross-flow filtration, and final formulation in Visipaque/Ringer solution. It results in 98% contaminating protein removal and 96% empty particle elimination. The final infectious particle concentration reaches 3.5E10 plaque forming units (PFU)/ml, with a specific activity of 6.8E11 PFU/mg protein. Overall recovery is over 40%. The newly established method is suitable for use in commercial production.

## Introduction

Parvovirus H-1 (H-1PV) belongs to the family *Parvoviridae* (Cotmore et al. 2014). It consists of a non-enveloped icosahedral capsid 25 nm in diameter, containing a linear ~5 kb single-stranded DNA genome. The viral DNA encodes non-structural proteins—notably NS1 (83 kDa) and NS2 (25 kDa)—and the capsid proteins VP1 (81 kDa) and VP2 (65 kDa). VP3 (63 kDa), another capsid protein, results from posttranslational cleavage of VP2, which takes place only in full particles (Faisst et al. 1995; Halder et al. 2012; Hanson and Rhode 1991). Although H-1PV is a rodent virus, it can infect and replicate in cells of a variety of vertebrates, including humans, and is known to exhibit oncolytic and oncosuppressive properties in a number of tumor models (Nuesch et al. 2012; Rommelaere et al. 2010). As a result of promising preclinical studies, the first in-man phase I/IIa clinical trial of H-1PV was conducted in 2011–2015 in patients with recurrent glioblastoma (Geletneky et al. 2012). At the moment, a second phase I/II clinical trial has been initiated in patients with inoperable metastatic pancreatic cancer (ClinicalTrials.gov Identifier: NCT02653313; manuscript in preparation).

For these trials, the H-1PV good manufacturing practice (GMP) batches were purified by density gradient centrifugation (Leuchs et al. 2016; Ungerechts et al. 2016); however, this method is limited in scale. Upscaling is needed for the next clinical trial phases. It is therefore necessary to establish a simple, efficient, robust, reproducible, and scalable chromatography-based purification process allowing elimination of empty capsids and protein impurities in one step, while promoting high recovery of infectious particles. Furthermore, purified H-1PV has ultimately to be formulated into Visipaque (48% iodixanol in Ringer solution), where the virus has known stability and which is also an X-ray contrast reagent for drug visualization in the human body.

We describe here a scalable, reproducible, high-grade chromatography-based procedure for H-1PV purification. An important challenge is to eliminate empty particles which were assembled but in which viral DNA failed to be packaged. The known difficulty of separating empty from full (genome-containing) particles by chromatography prompted us to first determine the isoelectric point difference between empty and full particles. In a second step, we exploited this difference to eliminate empty particles as well as protein contaminants by anion exchange chromatography, thus obtaining highly pure batches of infectious particles. Convective interaction media (CIM^®^)diethylaminoethyl (DEAE) monolith columns were used, as they achieve convective mass transport resulting in a high flow rate, yield, and efficiency. They also capture large molecules or particles such as H-1PV. What is more, these columns do not lose resolution upon upscaling (Rajamanickam et al. 2015).

## Materials and methods

### Preparation of clarified H-1PV cell lysate

H-1PV wild-type (wt) virus was produced in a 10-layer CellSTACK^®^ (CS; Corning, Wiesbaden, Germany) according to Leuchs et al. 2016. For this, NB-324K cells were seeded at 3.6E4 cells/cm^2^ and infected immediately with H-1PV wt (Kestler et al. 1999) at a multiplicity of infection (MOI) of 0.01 plaque forming units (PFU) per cell. To generate the clarified cell lysate, the cells were harvested after 4 days of incubation, lysed in 50 mM Tris-HCl (Trizma^®^ hydrochloride; Sigma-Aldrich Co, St. Louis, USA) buffer, DNAse-treated (50 U/ml, Sigma-Aldrich Chemie GmbH, Steinheim, Germany), and filtered through 0.2- and 0.1-μm membranes (Sartorius AG, Göttingen, Germany).

The H-1PV is also publically available at ATCC^®^ VR-356.

### Purification of H-1PV

#### IOD-PBS and VIS-Ringer density gradients

Iodixanol-PBS (IOD-PBS) and Visipaque-Ringer (VIS-Ringer) density gradient purifications were performed according to Leuchs et al. 2016 with 20 ml clarified cell lysate.

#### CsCl density gradient

CsCl density gradient purification was performed according to Leuchs et al. 2016.

#### Small- and large-scale DEAE anion exchange chromatography (AEX)

All chromatographic studies were performed with a 0.34-ml CIM^®^ DEAE monolith disk or an 8-ml CIM^®^ DEAE monolith column (1.3 μm pore size; Bia Separations, Ajdovscina, Slovenia) and ÄKTAprime (GE Healthcare Europe GmbH, Freiburg, Germany) at room temperature (RT) under laminar flow. The CIM^®^ monolith is a single homogeneous piece cast from methacrylate polymers containing flow through pores. DEAE is a weak anion exchanger with a charged diethylamino group which selectively binds molecules with a predominant negative charge. The flow rate of the mobile phase was 0.7 or 2 ml/min depending on the column size. Absorbance was monitored at 280 nm and is expressed in milli absorbance units (mAu). Conductivity is expressed in millisiemens (mS). All buffers and the sample were filtered through 0.2-μm filters. The column was equilibrated with the application buffer prior to the start of each run, until constant conductivity and UV absorbance values were observed. Elution steps were followed by a high-salt wash with 1 M NaCl (Sigma-Aldrich Chemie GmbH, Steinheim, Germany). The application buffer was 50 mM Tris-HCl or 0.15 M NaCl in 50 mM Tris-HCl, adjusted to pH 8.7. The sample was diluted in application buffer and applied to the column. This was followed by a wash with application buffer until baseline UV absorbance was reached. Elution was performed with a continuous salt gradient from 0 to 0.5 M NaCl in 50 mM Tris-HCl pH 8.7 or from 0.15 to 0.4 M (0.3 M for large scale) NaCl in 50 mM Tris-HCl pH 8.7. One-milliliter fractions were collected during the runs and analyzed for genome-containing particles (GP), physical particles (PP), and plaque forming units. Because plaque forming unit evaluation is a laborious cell-based method, it was done only in large-scale chromatographic experiments and for analysis of the final formulation, given the importance of the infectious virus titer in anticancer virotherapy.

### Formulation of purified H-1PV after the preparative DEAE column

The H-1PV eluate from the DEAE column contained 0.25 M NaCl. To eliminate salt and formulate into Visipaque (48% iodixanol in Ringer), it was necessary to perform buffer exchange into Ringer solution (AlleMan Pharma GmbH, Reutlingen, Germany) followed by formulation in Visipaque (48% iodixanol in Ringer).

For buffer exchange into Ringer solution, two approaches were used:

On the one hand, a 5-ml HiTrap™ desalting column (GE Healthcare Europe GmbH, Freiburg, Germany) was used with the ÄKTAprime system. HiTrap™ utilizes cross-linked dextran for size exclusion chromatography. The column was equilibrated first with five column volumes (CV) of Ringer solution. Then, the sample was injected into a 5-ml sample loop filled with Ringer solution, loaded onto the column, and eluted with two CV of Ringer solution. One-milliliter fractions were collected and analyzed for genome-containing particles and physical particles. On the other hand, 6 ml Vivaspin^®^ concentrators (cutoff 30 kDa; Sartorius AG, Göttingen, Germany) were used. The membrane ultrafiltration system was first sterilized with 70% ethanol for 20 min, centrifuged at 3000×*g*, rinsed with 6 ml sterile water for injection use, centrifuged again, and allowed to dry under laminar flow. Samples with a starting volume of about 1 ml were pipetted into the concentrator, diluted 1:5 with Ringer solution, and centrifuged at 3000×*g* until the volume reached 1 ml. Samples with a starting volume of 2 ml or more were first concentrated to 1 ml and then diluted with Ringer solution. This was done three times so as to dilute the initial buffer 1:125 in Ringer solution. The last concentration step was carried out down to a sample volume of ~300 μl. The refraction index of the sample was then measured with a digital refractometer AR200 (Reichert, Inc., Depew, NY, USA) and had to be that of Ringer solution (1.3342 ± 0.0002).

The samples in Ringer solution obtained by one of the above methods were mixed with Visipaque™ 320 (contains 65.2% iodixanol; GE Healthcare Europe GmbH, Freiburg, Germany) to 48% iodixanol final concentration. The refraction index of the sample was then measured and had to be that of a 48% Visipaque solution (1.41 ± 0.005).

### Quantification and qualification of H-1PV batches

Virus quantification and characterization were done by a plaque formation assay (for infectious particles), qPCR (for GP), H-1PV Capsid-ELISA (for PP), protein quantification and sodium dodecyl sulfate-polyacrylamide gel electrophoresis (SDS-PAGE) with silver staining, western blotting, and sterility assessment (see Leuchs et al. 2016 for method description).

#### Electron microscopy

Electron microscopic analysis of the purified virus batches was performed according to Leuchs et al. 2016 with a few minor modifications. A preincubation step with 0.05% BSA solution for 1 min was added before sample incubation as well as a virus deactivation step with 0.1% glutaraldehyde for 5 min after sample incubation. Photos were taken with a Zeiss EM 900 transmission electron microscope (Carl Zeiss Microscopy GmbH, Jena, Germany) at 85,000× magnification.

### Determination of H-1PV pI

#### Isoelectric focusing

Full and empty viral capsid preparations (CsCl density gradient purified) were separated in the electric field on the basis of their different isoelectric points (pI values).

All materials used for isoelectric focusing were purchased from Serva Electrophoresis GmbH, Heidelberg, Germany. The 0.9% agarose gels were cast on a GelBond film with added 2.5% Servalyt carrier ampholytes pH 5–8 forming a pH gradient in an electric field. Gel electrophoresis was performed with the electrophoresis power supply EPS 3500 XL (Amersham Pharmacia Biotech Europe GmbH, Freiburg im Breisgau, Germany) under laminar flow. The system was cooled to 10 °C, and the cooling reagent Bayol F was applied to the ceramic cooling plate between the gel and the plate. Filter paper strips soaked in electrode solutions were applied between the gel and the electrodes to maintain a stable gradient. An acidic solution was used at the anode and a basic one at the cathode. A 10-μl sample was placed on the gel surface by means of a silicon applicator strip with sample holes, laid on the gel surface. Separation conditions were as follows: isoelectric focusing of the sample at 250 V, 10 mA, 10 W for 10 min; 500 V, 10 mA, 10 W for 20 min; and 1000 V, 10 mA, 10 W for 40 min. Fixation and silver staining were performed according to Willoughby and Lambert 1983.

#### Chromatofocusing

Chromatofocusing is a protein separation technique in which proteins elute from the column according to their pI. Chromatofocusing of empty and full capsid virus preparations (CsCl density gradient purified) was done at RT under laminar flow with a Mono P 5/50 column (GE Healthcare Europe GmbH, Freiburg, Germany) and an ÄKTAprime equipped with a 500-μl injection loop. Mono P is a weak anion exchanger charged with mixed quaternary and tertiary amines. The flow rate of the mobile phase was 0.7 ml/min. The absorbance was monitored at 280 nm. All buffers and the sample were filtered through 0.2-μm filters. The application buffer was 0.025 M triethanolamine (Sigma-Aldrich Chemie GmbH, Steinheim, Germany) adjusted to pH 8.3 (for full and empty preparations) or 9.5 (for unpurified cell lysate) with concentrated iminodiacetic acid (Santa Cruz Biotechnology, Dallas, USA). The elution buffer consisted of Polybuffer 96 and Polybuffer 74 (GE Healthcare Europe GmbH, Freiburg, Germany) was diluted according the manual’s instruction in water for injection use, and the pH was adjusted to 5.0 (for full and empty preparations) or 3.5 (for unpurified cell lysate) with concentrated iminodiacetic acid. The descending linear pH gradients (pH 8 to 5 for full and empty particle preparations and pH 9.4 to 3.5 for unpurified cell lysate) were obtained according to Mono P 5/50 manufacturer’s instructions. For this, the column was equilibrated with application buffer at a pH slightly above the highest pH required. The elution buffer (adjusted to the lowest pH required) was passed through the column to generate first a pre-gradient. This was followed by application of the sample diluted in application buffer. Further application of the elution buffer to the column resulted in a moving descending pH gradient. This elution procedure led to formation of a pH gradient from pH 8.0 to 5.0 or from 9.4 to 3.5 with a Δ pH of 0.1–0.3 between fractions. Fractions of 0.5 or 1 ml were collected and analyzed for GP and PP. The pH of the collected fractions was measured manually with a Mettler Toledo InLab Viscous Pro pH electrode.

## Results

The aim of this study was to develop a purification process allowing separation of empty particles from particles containing a full genome and compatible with large-scale H-1PV production for clinical applications.

### Isoelectric point of H-1PV and process impurities

**Fig. 1:**
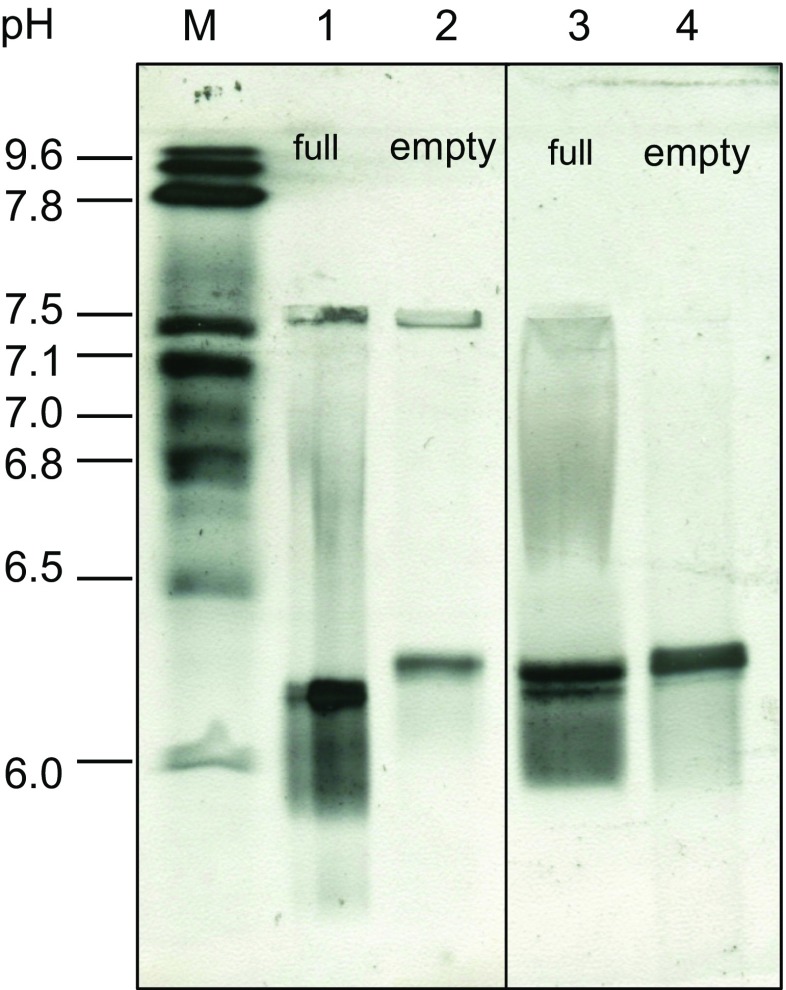
Determination of the isoelectric points of H-1PV wt empty and full capsids by isoelectric focusing. Two independently purified batches of empty or full capsids were loaded on the 0.9% agarose gel and focused in the electric field according to their respective pI values. The bands were visualized by silver staining. *M* indicates Biorad markers pH 4.45–9.6, *lane 1* indicates full particles from batch 1, *lane 2* indicates empty particles from batch 1, *lane 3* indicates full particles from batch 2, and *lane 4* indicates empty particles from batch 2

**Fig. 2:**
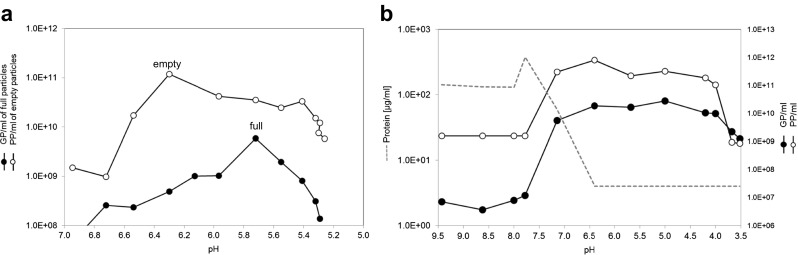
pI profiles of empty and full H-1PV capsids (**a**) and impurities (**b**), obtained by chromatofocusing with a Mono P 5/50 column. The fractions were analyzed for genome-containing particles (*GP*), physical particles (*PP*), and protein content (protein impurities). **a** The isoelectric point of empty capsid particles is at pH 6.3, as depicted by the physical particle peak and as evidenced by the PP-to-GP ratio (not shown): ratio ~869 at the pH corresponding to the PP peak. The isoelectric point of full capsid particles was around pH 5.6-6.1. The PP-to-GP ratio (not shown) was near 1 in this pH range. **b** The impurities in the clarified cell lysate (host cell proteins, FBS) showed isoelectric points between pH 9.5 and 7.0, eluting from the Mono P column before pH 7.0

In order to separate empty from full H-1PV wt viral particles, it was necessary to find an exploitable difference in their physical properties. Isoelectric focusing was performed on purified empty and full particle preparations to determine the respective isoelectric points of these particles. The results (Fig. 1) revealed a pI of 6.3 for empty particles and of 5.8–6.2 for full particles (with negligible contamination by empty capsids). To see if this pI difference might be exploited in purifying infectious virus (i.e., to eliminate empty particles and contaminating proteins), chromatofocusing was used. First, CsCl-purified empty or full particle preparations were loaded onto a Mono P 5/50 column and eluted as described under “Materials and methods”. By this procedure (Fig. 2a), chromatofocusing identified a pI of 6.3 for empty particles (PP-to-GP ratio ~869; data not shown) and of 5.8–6.1 for full particles (PP-to-GP ratio ~1; data not shown), thus confirming the results obtained by isoelectric focusing. Furthermore, chromatofocusing was used to estimate the pI range of impurities (host cell proteins, FBS) present in the clarified cell lysate. A significant amount of impurities were eluted from the Mono P column at basic pH (pI range 9.4 to 7). The PP-to-GP ratio was 43 at pH 6.4 versus 13 at near pH 5.7, in keeping with the pI values determined for empty and full particles.

### Small-scale anion exchange (AEX) chromatography for determining optimal buffer and salt conditions

The isoelectric points of proteins correlate with the charge at which they bind to/elute from the chromatography column. As the salt concentration increases, proteins with the weakest ionic interactions elute from the column first. With knowledge of the pI values of empty and full H-1PV particles and of the impurities arising during production, it is possible to separate these entities by AEX chromatography: most protein impurities should be eluted first, followed by empty particles and finally full particles.

As equilibration buffer for the AEX chromatography, 50 mM Tris-HCl pH 8.7 was chosen for its compatibility with virus harvest. For optimal binding, in theory, the pH of the buffer has to be at least 1–2 pH units above the pI of the protein (Fekete et al. 2015). At pH 8.7, the impurities should not bind, as they are weakly negatively charged, neutral, or even positively charged (pI between pH 9.5 and 7.0). Since the pI is 6.3 for H-1PV empty particles and 5.8–6.1 for full particles, the virus is negatively charged at pH 8.7. Preliminary studies performed with different ion exchange columns to determine binding and elution conditions (data not shown) demonstrated that a weak anion exchanger (DEAE) column is the best choice for H-1PV purification. A CIM^®^ DEAE monolith column with a 1.3 μm pore size was chosen for the chromatographic studies because of its ability to accommodate large molecules specifically while maintaining good mechanical stability.

**Fig. 3:**
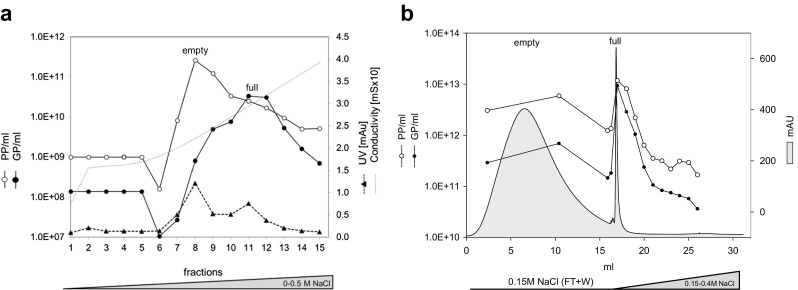
Optimal buffer and salt conditions for DEAE chromatography. **a** Pre-purified empty and full H-1PV particles in 50 mM Tris-HCl pH 8.7 were applied to the DEAE column and eluted with a continuous 0–0.5 M NaCl gradient. The elution profile shows the elution of empty capsid particles in fraction 8 (first UV absorbance peak) with 0.15 M NaCl (conductivity 22 mS). The second UV absorbance peak, starting at fraction 11, shows desorption of full capsid particles at 0.2–0.25 M NaCl (conductivity 29–32 mS). Physical particles (*PP*) and genome-containing particles (*GP*) were quantitated. **b** Clarified cell lysate was diluted in 50 mM Tris-HCl pH 8.7 containing 0.15 M NaCl and applied to the DEAE column. Empty particles were recovered in the flow through and in the 0.15 M wash (first UV absorbance peak). Upon elution with a 0.15–0.4 M NaCl gradient, full particles were eluted near 0.25 M NaCl (second UV absorbance peak). Physical particles (*PP*) and genome-containing particles (*GP*) were quantitated

Sodium chloride, sodium acetate, and ammonium acetate were tested to determine the best salt for elution. A mixture of pre-purified empty and full particles was applied to a 0.34-ml CIM^®^ DEAE monolith disk and eluted with a continuous 0–0.5 M gradient of the tested salt in 50 mM Tris-HCl pH 8.7. The peak fractions were analyzed for their PP and GP content. All elution profiles displayed two UV absorbance peaks: one corresponding to empty particles followed by one corresponding to full particles. As all the salts tested proved suitable for separating empty from full particles, we chose to continue working with NaCl because H-1PV is stable in NaCl solution (data not shown) and because NaCl is present in the desired final Visipaque/Ringer formulation (0.053 M NaCl). When a continuous gradient of NaCl was applied (0–0.5 M NaCl in 50 mM Tris-HCl pH 8.7), the empty particles were eluted at 0.13–0.15 M NaCl (PP-to-GP ratio ~300) and full particles at 0.2–0.25 M NaCl (PP-to-GP ratio ~1) (Fig. 3a). As these salt concentrations could not be measured directly, the corresponding conductivity values were also measured (18–22 mS for empty particles and over 25 mS for full particles) to ensure a reproducible purification process in further development experiments.

In a subsequent experiment, a small amount of clarified H-1PV harvest was prepared in 50 mM Tris-HCl pH 8.7 with 0.15 M NaCl and loaded onto the 0.34-ml CIM^®^ DEAE monolith disk to allow flow through of empty particles as well as impurities and binding of full particles to the column. The disk was then eluted with a 0.15–0.4 M NaCl gradient. As shown in the chromatogram of Fig. 3b, about 70% of the empty particles and 60% of the protein impurities were eliminated in the flow through and wash (first UV peak), whereas about 60% of the full particles were eluted near 0.25 M NaCl (second UV peak).

### Reproduced large-scale single step AEX chromatography

**Fig. 4:**
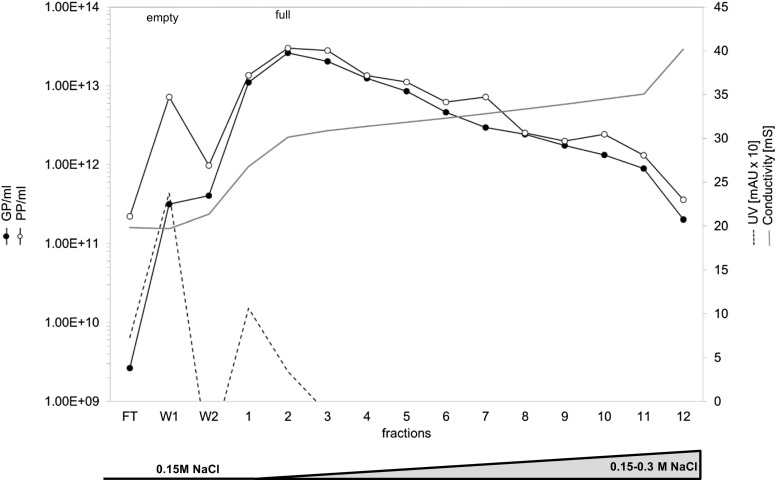
Large-scale chromatography of a 2.3E8 cell harvest (corresponding to one 10-layer CellSTACK®, reproduced five times). When clarified cell lysate diluted in 50 mM Tris-HCl pH 8.7 containing 0.15 M NaCl was loaded onto the 8-ml DEAE column, empty capsids was eluted in the flow through and wash (first UV peak). Full capsids was eluted in fractions 1–5 at NaCl concentration 0.25 M during elution with a continuous 0.15–0.3 M NaCl gradient (second UV peak, PP/GP ratio ~1)

After determination of the optimal buffer and salt conditions, a large-scale purification was performed with clarified cell lysate from 2.3E8 cells (corresponding to the production of one 10-layer CS). Clarified cell lysate diluted in 50 mM Tris-HCl pH 8.7 containing 0.15 M NaCl was loaded onto an 8-ml CIM^®^ DEAE monolith column and eluted with a continuous salt gradient (0.15–0.3 M NaCl in 50 mM Tris-HCl pH 8.7). As seen in Fig. 4, empty particles were eluted in the flow through and wash (first UV peak) (PP-to-GP ratio near 50.0; 2.3E14 total PP). Full particles were eluted at 0.2–0.25 M NaCl (conductivity 26–31.8 mS, second UV peak). The virus concentration was approximately 1E13 GP/ml and 1E10 PFU/ml (data not shown) and the PP-to-GP ratio was about 1.

Chromatographic purification of clarified H-1PV cell lysate was reproduced for five individual batches. These experiments revealed elimination of 67 ± 21% protein impurities and 65 ± 28% empty particles, with recovery of 72 ± 13% GP and 49 ± 13% PFU (Table 1). The salt concentration required for full infectious particles was 0.2–0.25 M NaCl, with a conductivity of 25–31.8 mS. Taken together, these results show that for the final formulation, it should be possible, in the future, to collect only one fraction, corresponding to this conductivity range.

**Table 1:** Comparison of five individual chromatography batches

Batch	Depletion of protein impurities [%]	Depletion of PP [%]	Elution of GP [%]	Elution of PFU recovery [%]
1	100	26	93	45
2	72	53	74	55
3	52	71	73	28
4	47	100	64	60
5	64	77	58	58
Mean [*n* = 5]	67 ± 21	65 ± 28	72 ± 13	49 ± 13

Means with standard deviations for five independent chromatography runs

### Buffer exchange and final formulation after AEX chromatography

**Fig. 5:**
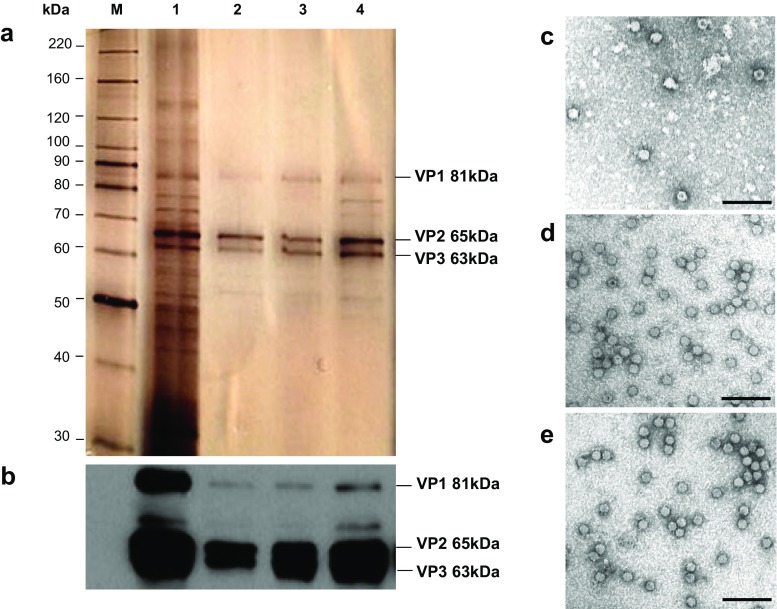
Protein composition and electron microscope images of virus batches before and after the downstream process. **a**, **b** Protein extracts of virus samples (1.0E10 PP) were analyzed by SDS-PAGE and revealed by **a** silver staining or **b** immunoblotting with αVP antibodies. *M* indicates markers constituting the BenchMark™ Protein Ladder, *lane 1* indicates virus harvest, *lane 2* indicates IOD-PBS-VIS-Ringer, *lane 3* indicates DEAE-VivaspinTM-final formulation, and *lane 4* indicates DEAE-HiTrap-final formulation. **c**, **d**, **e** Electron micrographs showing clarified cell lysate (**c**), IOD-PBS-VIS-Ringer (**d**), DEAE-final formulation (**e**). *Scale bar* is 100 nm

**Fig. 6:**
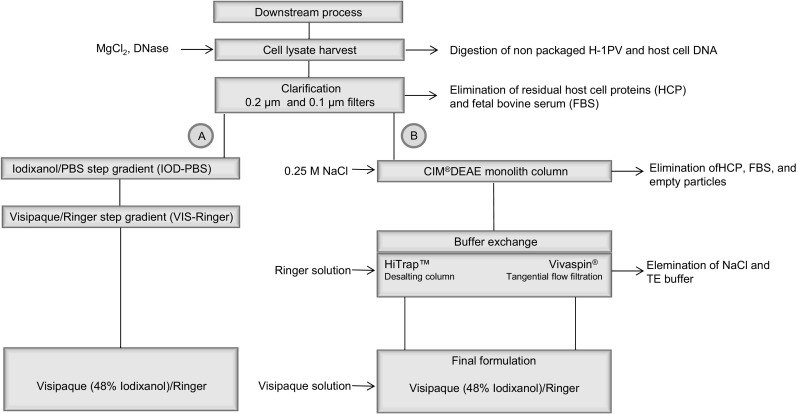
Flow diagram of H-1PV downstream purification methods, comparing IOD-PBS-VIS-Ringer gradient purification with DEAE-final formulation methods

For the final formulation in Visipaque/Ringer solution, the full particle eluate from the DEAE column had first to be buffer-exchanged into Ringer solution and finally mixed with Visipaque to obtain a refraction index of 1.41 ± 0.05, corresponding to 48% iodixanol. Two buffer exchange methods were tested: use of a HiTrap™ desalting column and use of Vivaspin^®^ concentrators. Each method was applied twice. The results were compared with those obtained by the standard purification method (density gradient centrifugation in IOD-PBS followed by VIS-Ringer) applied to the same virus harvest batch. Table 2 summarizes the results. With the newly developed chromatographic purification method, a preparation containing only full particles was obtained (PP-to-GP ratio ~1). PFU recovery was at least twice as high (around 40%) as with the standard method, and the specific activity was similar (or even higher after Vivaspin^®^ cross-flow filtration). The infectious virus titer obtained either by the standard method or by DEAE chromatography with Vivaspin^®^ filtration was about 3E10 PFU/ml; after DEAE chromatography and HiTrap™ desalting, it was 3E9 PFU/ml. The three methods reduced protein impurities with equal effectiveness (>96%), but chromatographic purification followed by either buffer exchange procedure eliminated empty particles more effectively (>95%) than the standard method (85%). Virus purity, as determined by SDS-PAGE with silver staining, was the same after all three procedures (Fig. 5). Electron micrographs of the preparations obtained by the novel strategy showed full particles predominantly, in contrast to standard preparations. The flow diagram in Fig. 6 summarizes the downstream process steps of all three methods. Quality control tests demonstrated that all final formulated batches were sterile.

**Table 2:** Comparison of H-1PV recovery and depletion of empty particles as well as protein impurities by chromatography- and density gradient ultracentrifugation-based downstream process from the same starting material

Method of purification	IOD-PBS-VIS-Ringer gradient	DEAE-Vivaspin^®^	DEAE-HiTrap™
Final formulation	Visipaque (48% iodixanol)/Ringer	Visipaque (48% iodixanol)/Ringer	Visipaque (48% iodixanol)/Ringer
PFU/ml	3.7 ± 0.6E+10	3.5 ± 0.5E+10	3.0 ± 0.3E+09
volume [ml]	1.3 ± 0.5	4.4 ± 0.2	39.1 ± 9.3
PFU/mg protein*	2.1 ± 0.1E+11	6.8 ± 2.5E+11	2.3 ± 0.3E11
Recovery PFU %*	13.1 ± 1.6	41.3 ± 15.5	42.3 ± 2.9
PP-to-GP ratio*	2.8 ± 0.5	1.1 ± 0.3	1.2 ± 0.0
Depletion of protein impurities %	98.5 ± 0.1	98.3 ± 0.3	96.5 ± 0.5
Depletion of empty particles %*	85.0 ± 1.3	95.8 ± 5.9	97.8 ± 1.5

Means with standard deviations (*n* = 2) of infectious titer (PFU/ml), recovery of infectious particles*, specific activity* (PFU/mg protein), the PP-to-GP ratio*, and depletion of empty particles* and protein impurities. The *p* value is ≤0.05 for the features marked with *asterisk*. For *p* value calculation, two further IOD-PBS-Vis-Ringer density gradient purified batches were taken into account

## Discussion

In this study, we have developed a simple, scalable high-grade purification method for H-1PV. Empty particles are an undesirable by-product of H-1PV production, liable to cause an immune response without efficacy (Gao et al. 2014). Although ultracentrifugation-based methods have been used successfully to eliminate empty particles (Halder et al. 2012; Leuchs et al. 2016), these procedures are not scalable, they are only semi-sterile, and fractionation is less controllable than with chromatographic methods. This explains why it was urgent to develop new purification approaches suitable for upscaling.

As a basis for chromatographic purification, we first determined the pI values of empty and full particles: 6.3 and 5.8–6.1, respectively. Isoelectric focusing and chromatofocusing gave similar results. For full particles, only a pI range could be obtained due to possible defective interfering virus particles (Faust and Ward 1979). Similar theoretical pI values were calculated for adeno-associated virus serotype 1 (pI, 6.3 for empty particles and 5.9 for full particles) (Venkatakrishnan et al. 2013). Knowledge of the isoelectric points of empty and full H-1PV was essential to developing the purification procedure and might have implications for virus interactions, stability, production, and in vitro studies (Xia 2007).

We have tested the ability of different chromatographic systems to eliminate empty particles and impurities. Unexpectedly, Capto Core 700, a chromatographic system combining size exclusion with anion exchange, failed to eliminate impurities under either acidic conditions (Ringer pH 5.6) or alkaline conditions (Ringer or 50 mM Tris-HCl, pH 8.7), as in both cases, most of the H-1PV was eluted at the cleaning-in-place step with 1 M salt wash or 1 M NaOH in 30% isopropanol. Slightly better results were obtained by applying H-1PV diluted in Ringer solution (pH 5.6): 17% full particle recovery in the flow through (data not shown). This is in disagreement with the column manufacturer’s claim that viruses and large molecules (M_r_ > 700 kDa) are collected in the flow through (GE Healthcare, Application note 29-0983-01 AB). The H-1PV capsid, with a molecular weight of around 6E6 Da, should theoretically pass through the column. The reason for this failure of Capto Core 700 is unknown and was not further investigated. Binding studies with H-1PV have demonstrated that the virus binds to and elutes from both cation and anion exchangers, but best recoveries were achieved with AEX chromatography and elution at pH 8.7 (data not shown). We were also able to purify H-1PV on a strong anion exchange column (QA), with similar full particle recovery (data not shown), but as no improvement over DEAE chromatography was observed in terms of recovery and protein impurity elimination, we decided to continue working with DEAE. Likewise, as sodium chloride, sodium acetate, and ammonium acetate in the eluent gave rise to similar resolution of the peaks corresponding to empty and full capsids, sodium chloride was chosen for H-1PV purification because H-1PV is stable in this salt and because NaCl is present in the final formulation.

The fact that the pI difference between full and empty particles is at least 0.1 pH unit has enabled us to develop a DEAE purification method. We show that 65% of the empty particles and 67% of the main impurities can already be eliminated in the flow through with 0.15 M NaCl, and that 50% of the full infectious virus particles can be eluted with 0.3 M salt. Thanks to the high stability of H-1PV, the purification can be performed at room temperature (operating temperature 29 ± 0.45 °C). Temperature, flow rate, and impurities influence the chromatography and should be taken into account for the reproducibility and robustness of the method (Mihelic et al. 2003; Acikara 2013).

With the present method and conditions, the H-1PV elution profile could not be reproduced with the closely related protoparvovirus minute virus of mice (MVM) (data not shown), and empty MVM particles could not be separated from full ones. The method as developed here is thus specific to H-1PV.

Although the present method offers an H-1PV yield of nearly 50% from the clarified cell lysate, one should note that the medium supernatant of the upstream process is still routinely discarded, amounting to a 30% loss (Leuchs et al. 2016). To demonstrate proof of concept and to test the ability of our method to purify and concentrate H-1PV, clarified medium supernatant from routine production (corresponding to 1/10 of a 10-layer CS production) was purified as described here. After DEAE purification, total PFU recovery was about 50%, as observed with cell lysate, but the impurity content of H-1PV purified from culture medium supernatant was still high. Therefore, a second round of DEAE purification was performed, yielding ~20% total PFU recovery in a 50th of the initial volume. Over 99.7% of the protein impurities were eliminated after the second DEAE purification step, so that the protein content was the same as for purified cell lysate (data not shown). Clearly, more optimization and upscaling will be required to include the medium supernatant in the complete downstream purification process in order to achieve a higher overall yield.

For the final formulation, both buffer exchange methods were suitable, but use of the Vivaspin^®^ tangential flow filtration system to exchange the buffer and simultaneously concentrate the sample to the desired volume is optimal with negligible loss. Buffer exchange with the HiTrap™ desalting column leads to sample dilution and thus to a lower infectious virus titer in the final formulation. Between the two buffer exchange methods, there is a one-log infectious virus titer difference in the final formulation (3.5E10 and 3.0E9 PFU/ml for Vivaspin® and HiTrap™ desalting, respectively).

The final novel downstream process, which achieves the target virus titer of 1E10 PFU/ml, involves the following steps: (1) DNase treatment to eliminate host cell DNA, (2) clarification (0.2 μm followed by 0.1 μm filtration steps), (3) anion exchange chromatography to eliminate empty particles and most impurities, (4) a tangential flow filtration for buffer exchange and concentration, and (5) final formulation in Visipaque/Ringer solution.

As compared to iodixanol-based ultracentrifugation, use of this novel purification strategy involving monolith-based DEAE anion exchange chromatography significantly improves recovery and specific activity, leading to an optimal PP-to-GP ratio and significant elimination of empty particles (*p* values ≤0.05). The performance of the new method is similar to that of the ultracentrifugation-based CsCl density gradient method, in terms of the PP-to-GP ratio and PFU per milligrams of protein (Leuchs et al. 2016). The virus titer of 1E10 PFU/ml is optimal, because the virus particles start to aggregate at 1E11 PFU/ml (as attested by high variance of the titer) and because preparations with a virus titer lower 1E8 PFU/ml are unstable over the years (data not shown). The overall recovery of 40% with our new purification strategy is comparable with or superior to those obtained in other virus/viral vector productions (Adamson-Small et al. 2016; Merten et al. 2016).

Upscaling for clinical applications is possible, with the larger CIM® DEAE monolith columns commercially available up to 8 l. These could be loaded with a total of 5E14 PFU, (corresponding to 1000 10-layer CSs). Monolithic columns do not lose resolution with scale-up, so there is no need for new characterization studies. CIM® DEAE monolith columns offer a high binding capacity with high flow rates and low pressure (Mihelic et al. 2000), which could significantly reduce purification time and costs. Furthermore, these columns can be used as disposables or for multiple uses and as a single column or in multicolumn setups. As the limiting loading capacity of an 8-ml column is 20 mg BSA/ml and as we loaded such a column with only ~15 mg total protein (from cell lysate corresponding to 2.3E8 cells totaling about 5E11 PFU), the column could be loaded with 10 times as much. Furthermore, the tangential flow filtration is easily upscalable by using the proven polyethersulfone membrane with a pore size of 30 kDa. For upscaling, it is nevertheless necessary to establish the optimal flow rates and pressure.

In summary, we have developed a simple, robust one-step chromatography-based purification strategy enabling elimination of empty H-1PV particles and most impurities on the basis of their isoelectric points. This novel method offers sufficient recovery and maintains viral infectivity. It will facilitate aseptic upscaling according to GMP guidelines for clinical applications.
